# Aedes aegypti mosquitoes from Burkina Faso can transmit the chikungunya virus and Zika virus of the African lineage, but not the Zika virus of the Asian lineage

**DOI:** 10.1099/jgv.0.002103

**Published:** 2025-06-19

**Authors:** Aboubakar Sanon, Lanjiao Wang, Athanase Badolo, Leen Delang

**Affiliations:** 1 Virus-Host Interactions & Therapeutic Approaches (VITA) Group, Department of Microbiology, Immunology and Transplantation, KU Leuven, Leuven, Belgium; 2 Laboratoire d’Entomologie Fondamentale et Appliquée, Université Joseph Ki-Zerbo, Ouagadougou, Burkina Faso

## Abstract

*Aedes aegypti* is the primary vector for the transmission of dengue virus (DENV), Zika virus (ZIKV), and chikungunya virus (CHIKV). While Burkina Faso has been facing an increase in DENV epidemics, no ZIKV or CHIKV outbreaks have been reported. Here, we, therefore, assessed the vector competence of *Ae. aegypti* mosquitoes from Burkina Faso for ZIKV and CHIKV transmission. *Ae. aegypti* mosquitoes from urban and peri-urban sites of Ouagadougou were orally infected via blood meal with different titres of a West African CHIKV strain and ZIKV strains of the Asian and African lineage. The infection rate for mosquitoes from both sites was ~65% for CHIKV, whereas the dissemination was 85% and 45%, respectively, in urban and peri-urban mosquitoes. The CHIKV transmission rates ranged between 14 and 18%. For the African lineage of ZIKV, the infection rate was higher for urban mosquitoes than peri-urban mosquitoes (100% vs. 80%), whereas the dissemination rate was 100% in mosquitoes from both sites. The transmission rates for African ZIKV ranged between 12 and 18% for both urban and peri-urban mosquitoes. In contrast to ZIKV of the African lineage, low infection rates (10–30%) and dissemination rates (14–50%) were observed with the Asian lineage of ZIKV. Additionally, mosquitoes from both sites could not transmit the ZIKV strain of the Asian lineage. This study demonstrates, for the first time, the capability of *Ae. aegypti* mosquitoes from Burkina Faso to transmit chikungunya virus and the pre-epidemic strain of Zika virus in their saliva, highlighting the importance of establishing surveillance for these viruses in Burkina Faso.

## Introduction

Arboviruses are prevalent in Africa, where they provoke serious epidemics and cause major public health challenges. However, the burden of arboviruses like dengue virus, Zika virus or chikungunya virus in Africa is often underreported or misdiagnosed as malaria. Chikungunya virus (CHIKV) and Zika virus (ZIKV) are two arboviruses that are transmitted by *Aedes aegypti* and *Aedes albopictus* mosquitoes [1]. CHIKV is a member of the *Alphavirus* genus in the *Togaviridae* family. This virus causes an acute illness characterized by fever and arthralgia, headaches, nausea and vomiting [2]. In a subgroup of patients, a CHIKV infection can evolve into a chronic disease, with recurrent episodes of severe joint pain. CHIKV was first isolated in Tanzania in 1952 from the serum of an infected patient [3]. The virus has caused several outbreaks in tropical Africa and Asia [4]. In 2013, CHIKV emerged in the Americas, resulting in a large outbreak with millions of people infected. In 2023, CHIKV caused more than 460,000 cases and 350 deaths were reported globally. In West Africa, 565 cases were confirmed across Senegal, Burkina Faso, Mali, The Gambia and Guinea by November 2023 [5,6]. Three lineages of CHIKV are circulating worldwide: the West African, Asian and East Central South African (ECSA) lineages. The latter includes the ECSA-derived Indian Ocean lineage [7].

ZIKV belongs to the *Flaviviridae* family of the genus *Orthoflavivirus* and was first isolated in East Africa in 1947 [8]. Decades later, ZIKV has spread from Africa to new territories in the Americas and the Pacific Islands, resulting in unprecedented outbreaks with millions of confirmed cases [9]. Two lineages of ZIKV have been previously described, which are present in two geographic regions: the African and the Asian lineage [10]. The Pacific and the American virus clades have been identified within the Asian lineage [11,12]. ZIKV infection is usually asymptomatic or causes mild disease with characteristic symptoms such as maculopapular rash, fever, arthralgia and conjunctivitis [13]. However**,** in recent epidemics, microcephaly [14,15] and central nervous malformations in neonates born from a ZIKV-infected mother, and Guillain–Barré syndrome [16] in ZIKV-infected adults, have been reported.

In West Africa, ZIKV has been found to circulate in several countries such as Senegal, Nigeria, Mali and Côte d’Ivoire through serology testing of human blood. In Senegal and Nigeria, ZIKV has also been isolated from human blood samples [17]. Additionally, ZIKV has been frequently detected in African mosquitoes [18,19]. Despite this, only a limited number of human cases have been reported in Africa so far, and no large epidemics of ZIKV have been observed. Previous studies have indicated that African mosquitoes are capable of transmitting ZIKV, but the susceptibility depends on the ZIKV lineage (Asian or African) and the domestication level of the *Ae. aegypti* population [20]. The domestic subspecies of *Ae. aegypti*, *Ae. aegypti aegypti (Aaa*), has been found to have a higher susceptibility to ZIKV compared to the African subspecies, *Ae. aegypti formosus* (*Aaf*). The difference in ZIKV susceptibility is most pronounced for Asian ZIKV strains and less evident for African ZIKV strains.

In Burkina Faso, serological surveillance in several districts of the capital city has shown that several flaviviruses circulate in humans [21]. Due to the cross-reactivity among flaviviruses in these tests, it is difficult to deduce which specific flaviviruses are present. A recent serological surveillance study for ZIKV revealed a higher prevalence of ZIKV antibodies in blood donors from Ouagadougou compared to those from Bobo-Dioulasso [22]. Other studies have detected the presence of dengue virus and CHIKV in *Ae. aegypti* mosquitoes from Burkina Faso and in humans [10,23, 24] through PCR and serology tests, respectively. Additionally, recent studies have reported CHIKV antibodies in human sera collected in Ouagadougou, suggesting the circulation of this virus. A sero-epidemiological reconstruction model indicated that CHIKV has been circulating unnoticed in the country for years [25]. Moreover, it was estimated that seven outbreaks of CHIKV have occurred in Ouagadougou since the 1970s (none since 2001), but none of these outbreaks were reported. Recently, in 2023, CHIKV infection has also been reported in patients [26,27]. In this study, we assessed the competence of two *Ae. aegypti* populations from the capital city of Burkina Faso to transmit ZIKV and CHIKV.

## Methods

### Mosquito collection and rearing

**Fig. 1.**
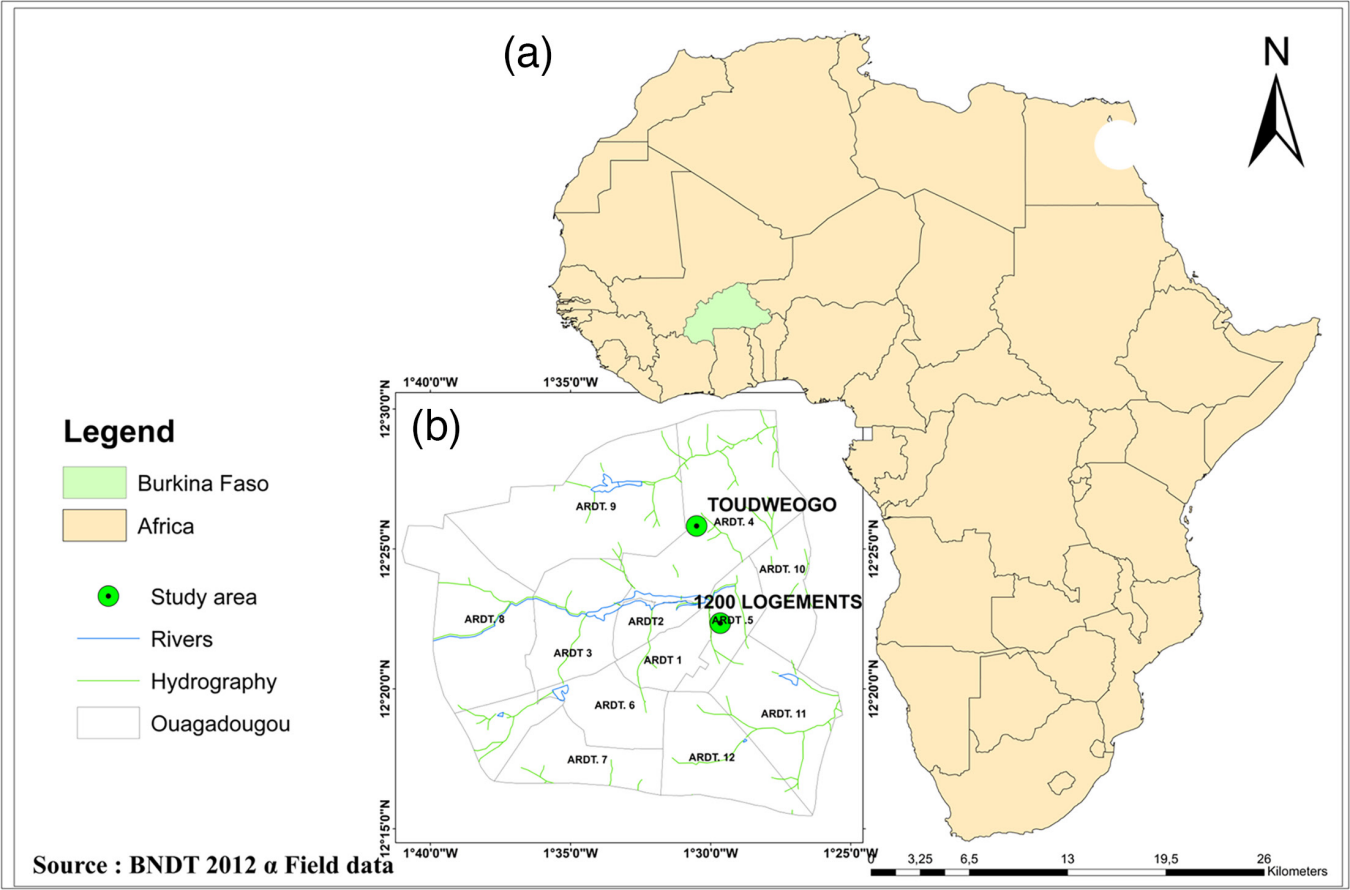
Map showing the geographical location of the collection sites. (**a**) The map of Africa showing Burkina Faso. (**b**) Localization of the exact sampling sites of mosquito samples. BNDT, Base Nationale des Données Topographiques.

Larvae of *Ae. aegypti* were collected from September to November 2022 from two sampling sites in Ouagadougou, the capital city of Burkina Faso (Fig. 1). The two sites were chosen based on the degree of urbanization. 1200 logements (1200LG) (12° 22′ N 1° 29′ W) is an urban site located 2 km from the international airport. The roads of this site are paved and lined by a channel that allows the evacuation of polluted water. Toudweogo (Toud) (12° 26′ N 001° 30′ W) is a peri-urban area located in the North of the capital city, 6 km from 1200LG. The vegetation of this site is sparse, with unpaved roads and a poor waste management system.

Mosquito larvae were collected from *Ae. aegypti* breeding sites such as tyres, flower pots and metal and plastic containers. These larvae were transported to the laboratory in Ouagadougou, where they were provided with cat food and reared until they emerged as adults. The adult mosquitoes were kept in the insectary at a temperature of 27±2°C and a relative humidity of 75±5%, and they were allowed to feed on a 10% sugar solution.

The adult mosquitoes were blood-fed on mice to produce the first and second generation (F1/F2) eggs. These eggs were hatched in the insectary at KU Leuven in dechlorinated tap water, and the resulting larvae were transferred to plastic trays containing 3 l of water. They were fed daily with a yeast tablet (Gayelord Hauser, Saint-Genis-Laval, France) until they reached the pupal stage. The pupae were then placed in small bowls and allowed to emerge inside 30×30×30 cm BugDorm cages (MegaView Science Co., Ltd., Taichung, Taiwan). The F2-F3 adult mosquitoes were provided with cotton pledgets soaked in a 10% sugar solution. The trays and cages were maintained at 28±1°C and 80 relative humidity with a light/dark cycle of 16:8 h.

### Viruses and cells

African green monkey kidney cells (Vero cells, ATCC CCL-81; Vero E6 cells, ATCC CRL-1586) were maintained in minimum essential medium (MEM 1×) supplemented with 10% FBS, 1% sodium bicarbonate, 1% l-glutamine and 1% non-essential amino acids (NEAA). Mammalian cell cultures were incubated at 37 °C with 5% CO_2_. C6/36 cells (larval cells of *A. albopictus*, ATCC CRL-1660) were cultured in Leibovitz’s L-15 medium supplemented with 10% FBS, 1% penicillin–streptomycin (PenStrep), 1% NEAA and 1% HEPES buffer. The cells were incubated at 28 °C without CO_2_. All cell culture media and supplements were obtained from Gibco™, Thermo Fisher Scientific (Aalst, Belgium).

The West African strain of the ZIKV (SEN-11, PRJEB39677) was initially isolated from mosquito pools in Kedougou in 2011 and kindly provided by Dr. Ousmane Faye of Institut Pasteur in Dakar. The epidemic Puerto Rico strain of the ZIKV (PRVABC59, KU501215) was isolated from a human serum specimen in Puerto Rico in 2015. The stocks of the ZIKV West African strain and Puerto Rico strain were produced on Vero E6 cells and C6/36 cells, respectively, and stored at −80 °C.

The West African CHIKV strain (CHIKV/1983/SN/WA 37997) was isolated in 1983 from *Aedes furcifer* mosquitoes in Senegal and acquired via EVAg. A stock of CHIKV was generated on C6/36 cells (passage 5) and stored at −80 °C.

### Oral infection of mosquitoes

To assess the vector competence of *Ae. aegypti* mosquitoes from Burkina Faso for CHIKV and ZIKV transmission, we evaluated 2 l of CHIKV (low: 1×10^7^ p.f.u. ml^−1^; medium, 2×10^8^ p.f.u. ml^−1^), 3 l of the ZIKV African strain (low, 1×10^7^; medium, 1×10^8^; and high, 1.4×10^9^ TCID_50_/ml) and 2 l of the ZIKV Asian strain (low, 1×10^7^; medium, 1×10^8^ TCID_50_/ml); titres mentioned are the final titres adjusted in the blood meal. Oral infection of mosquitoes was performed in a climate chamber at 27 °C and 75–80% humidity using a Hemotek apparatus (Hemotek Ltd, Blackburn, UK). Briefly, 7–10-day-old mosquitoes were sugar-starved for 24 h. The mosquitoes were given an artificial blood meal containing rabbit erythrocytes, adenosine triphosphate (ATP, 5 mM; Cayman Chemical, USA) and either CHIKV or ZIKV. Following feeding, fully engorged mosquitoes were cold-anesthetized and placed in cardboard cups with access to a 10% sucrose solution in the climate chamber set at a temperature of 28 °C, 80% relative humidity, and a 16:8 h light/dark cycle. Mosquitoes were sacrificed for virus detection at 7 and 14 days post-infection for CHIKV and ZIKV, respectively. The different time points were selected according to the extrinsic incubation period of each virus, which corresponds to the time between the infection and the presence of the virus in the saliva.

Mosquito saliva and body parts were collected as previously described [28]. Briefly, mosquitoes were sugar-starved for 24 h prior to salivation. Wings and legs were removed using sterile forceps. The proboscis of each mosquito was inserted into a pipette tip containing 20 µl of FBS to allow salivation for 1 h. Each saliva sample was mixed with 20 µl of Dulbecco's Modified Eagle Medium (DMEM) supplemented with 2% FBS and 1% PenStrep. The head, wings and legs, as well as the body of each mosquito, were separated using fine sterile forceps and placed in individual homogenization tubes containing ceramic beads. The wings and legs of each mosquito were grouped together with the head sample. All samples were stored at −80 °C until further use.

The heads, wings and legs, as well as the bodies, were diluted in 500 µl PBS, homogenized using bead disruption and filtered through 0.8 µm MINI column filters to remove debris, bacteria and fungi. The filtered homogenate was used for endpoint dilution assays to determine and quantify infectious virus titres.

### Determination of virus titres in mosquito samples

#### Endpoint titrations

Vero and Vero E6 cells were seeded at a density of 2.5×10^4^ and 10^4^ cells in 96-well tissue culture plates for CHIKV and ZIKV samples, respectively, and allowed to adhere overnight at 37 °C. The next day, a 10-fold dilution series of homogenates of the mosquito bodies or the head, wings and legs was added to the cells. After 3 and 6 days of incubation at 37 °C for CHIKV and ZIKV, the plates were examined under the microscope for signs of virus-induced cytopathic effect. The virus titre was quantified as TCID_50_/ml and determined using the Reed and Muench method [29].

#### Plaque assay

Vero and Vero E6 cells were seeded in 6-well tissue culture plates at a density of 10×10^6^ cells/well in 10% medium. The next day, the medium was aspirated, and the cells were washed with 1 ml of 2% assay medium. Subsequently, the cells were incubated with specified virus dilutions in 2% medium for 1 h at 37 °C. After the incubation period, the virus was removed, and the cells were washed three times with 1 ml of 2% assay medium. Then, a freshly prepared 1:1 mixture of 1% low-melting agarose (Invitrogen, Belgium) and 2xMEM medium (Gibco, Belgium) was added. After 3 and 6 days of incubation for CHIKV and ZIKV, respectively, the cells were fixed with 3.7% formaldehyde, followed by the removal of the carboxyl-methyl cellulose overlay, and the cells were stained with crystal violet staining solution to visualize virus plaques and determine the infectious virus titre (p.f.u.).

#### Statistical analysis

GraphPad Prism (9.5.1) was used to generate graphs and to analyse the datasets statistically. The infection rate (number of positive bodies/numbers of fed mosquitoes tested), the disseminated infection rate (number of mosquitoes with positive heads–wings–legs/number of mosquitoes with positive bodies), the transmission rate (number of positive saliva/number of mosquitoes with positive heads–wings–legs) and the transmission efficiency (number of positive saliva/numbers of fed mosquitoes tested) for each virus and each collection site were calculated. These infection, dissemination and transmission rates, as well as the transmission efficiency, were statistically compared using the Fisher test. The viral loads were statistically analysed using the Mann–Whitney *U* test (urban vs peri-urban), and the statistical threshold was set at *P*<0.05.

## Results

### *Ae. aegypti* from Burkina Faso are competent for CHIKV transmission

**Fig. 2.**
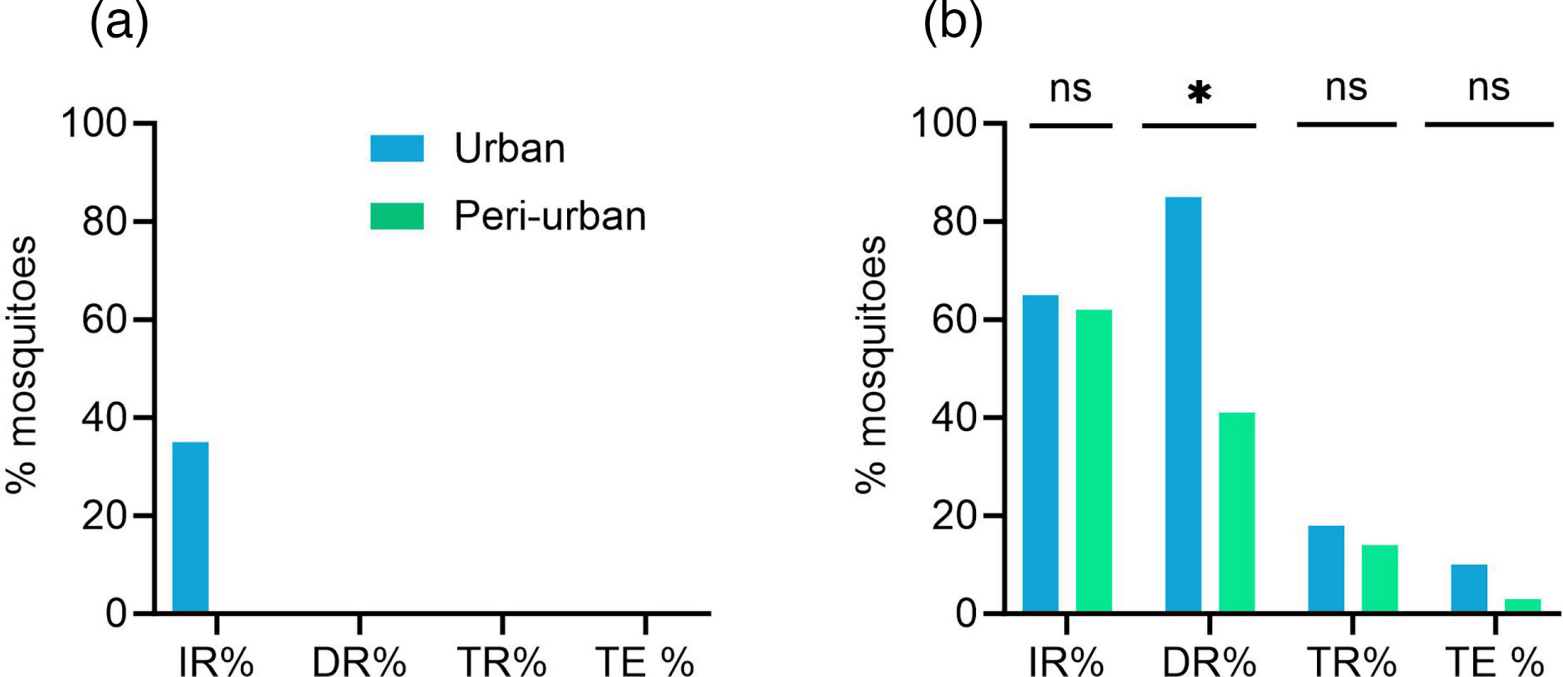
CHIKV infection rate (IR), dissemination rate (DR), transmission rate (TR) and transmission efficiency (TE) of *Ae. aegypti* from the urban (blue) and peri-urban (green) sites for (**a**) a low titre of 10^7^ p.f.u. ml^−1^ and (**b**) a medium titre of 2×10^8^ p.f.u. ml^−1^. The differences between the two groups (urban vs. peri-urban) were analysed with the Fisher exact test (ns, *P*>0.05; *, *P*<0.05).

Two mosquito populations from urban and peri-urban sites in Ouagadougou were orally infected with chikungunya virus using two different virus inocula in the blood meal. For the low titre of 10^7^ p.f.u. ml^−1^, the infection rate of mosquitoes from the urban site was 35%, but the virus did not disseminate (Fig. 2a and Table S1, available in the online Supplementary Material). Mosquitoes from the peri-urban site did not get infected. In contrast, when exposed to the medium virus titre (2×10^8^ p.f.u. ml^−1^), ~65% of the mosquitoes were infected, with no significant difference observed between mosquitoes from the urban and peri-urban sites (Fig. 2b and Table S1). However, the dissemination of CHIKV differed significantly between mosquitoes from both sites (85% vs 41% for urban and peri-urban sites) (Fig. 2b). Both mosquito populations were able to transmit CHIKV in their saliva, with transmission rates ranging from 1418%.

**Fig. 3.**
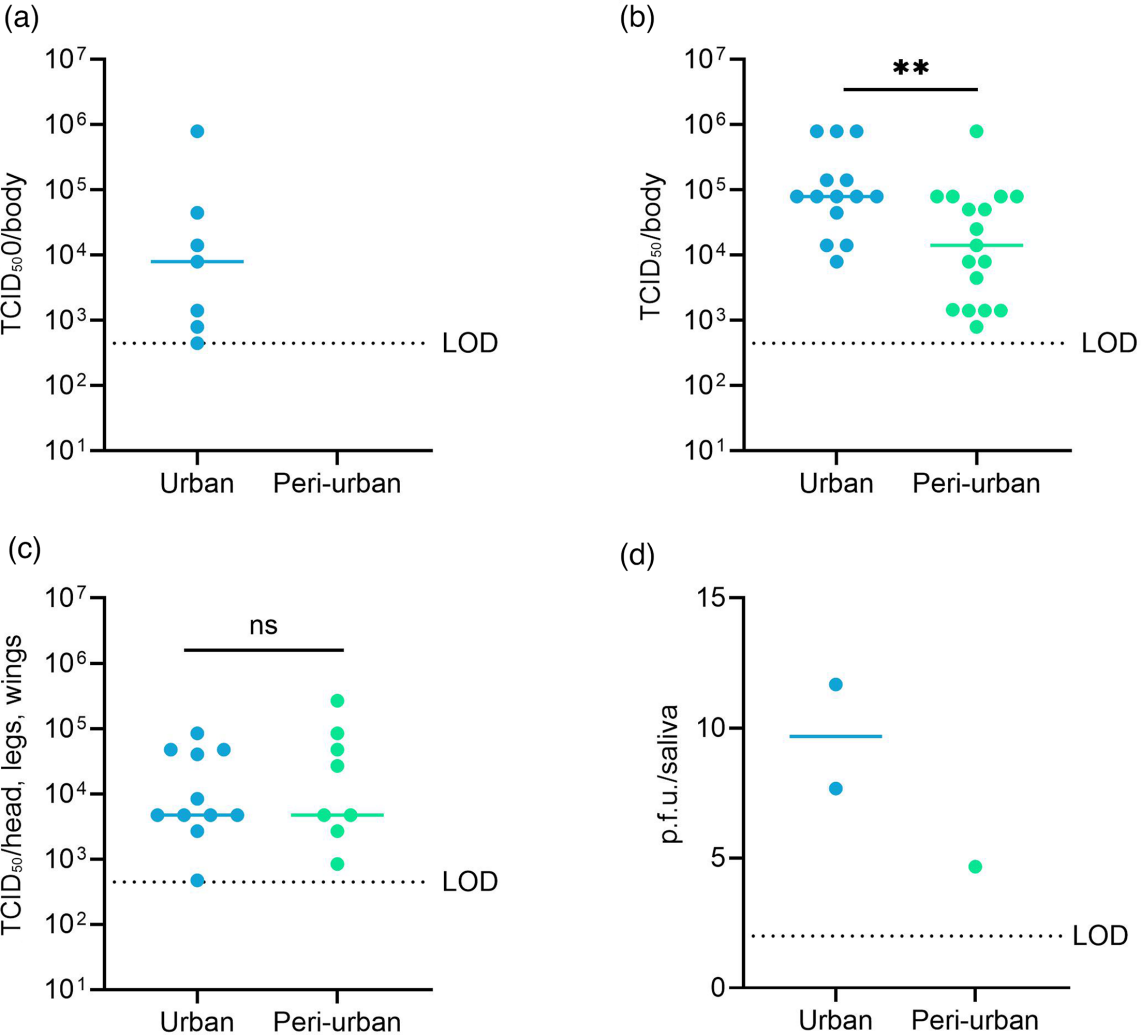
CHIKV titres in *Ae. aegypti* bodies infected with a low titre of 10^7^ p.f.u. ml^−1^ (**a**) and CHIKV titres in bodies (**b**); heads, legs, wings (**c**); and saliva (**d**) of mosquitoes infected with 2×10^8^ p.f.u. ml^−1^. CHIKV titres were quantified by endpoint titration (bodies and head) and plaque assay (saliva). The viral loads in the different parts of the mosquito have been quantified at 7 days post-infection for CHIKV. Each point on the plot represents an individual mosquito. All plots show the median value. LOD is the limit of detection. Statistical analysis was performed with the Mann–Whitney test; ns, *P*>0.05; **P<0.005.

After exposure to the low titre of 10^7^ p.f.u. ml^−1^, the median CHIKV titre in the body was 7.9×10^3^ (95% confindence interval (CI): 4.45×10^2^ – 7.90×10^5^) TCID_50_/sample for the urban site, while no virus was detected in mosquitoes from the peri-urban site (Fig. 3a). The median CHIKV titres detected in mosquito bodies after exposure to the medium titre were 7.9×10^4^ (95% CI: 1.4×10^4^ – 7.9×10^5^) and 1.4×104 (95% CI: 1.4×10^3^ – 7.9×10^5^) TCID_50_/body for mosquitoes of the urban and peri-urban sites (Fig. 3b). In the heads/legs/wings, median CHIKV titres were observed of 4.7×10^3^ (95% CI: 2.7×10^3^ – 4.7×10^4^) and 4.7×10^3^ (95% CI: 0–8.4×10^4^) TCID_50_/sample for urban and peri-urban mosquitoes, respectively (Fig. 3c). The number of infectious CHIKV particles detected in the saliva was low: 9.7 and 4.7 p.f.u. per saliva sample for mosquitoes of the urban and peri-urban sites (Fig. 3d). For mosquitoes exposed to the medium virus titre, the viral loads varied significantly in mosquito bodies, but not in the heads/legs/wings between mosquitoes from the urban and peri-urban sites (Fig. 3b,c).

### *Ae. aegypti* from Burkina Faso can transmit the Zika virus from the African lineage

**Fig. 4.**
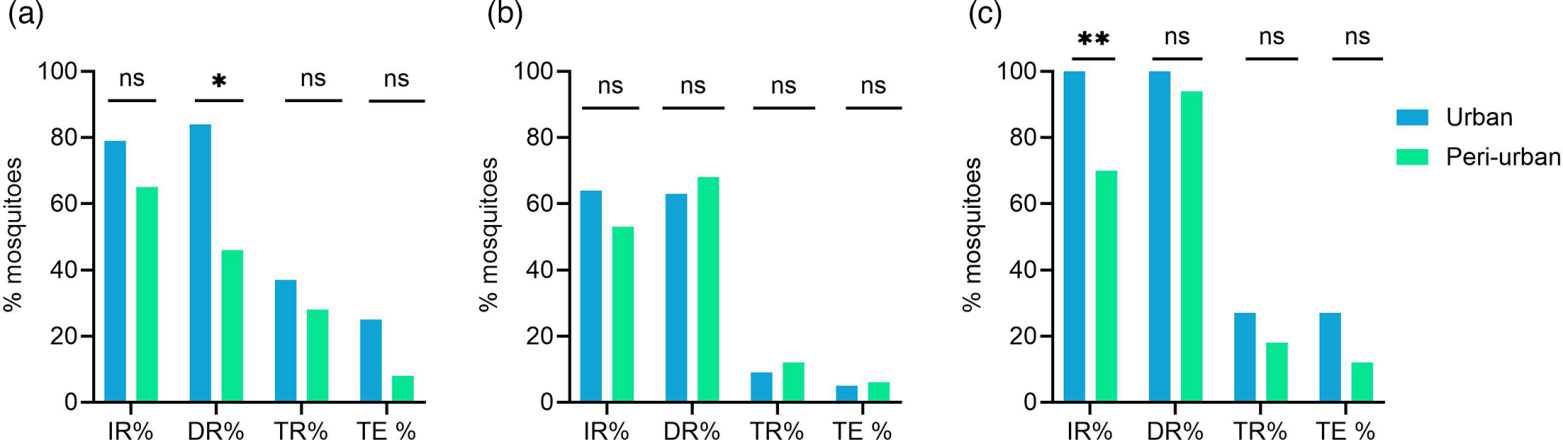
ZIKV IR, DR, TR and TE of urban and peri-urban mosquitoes infected with a low titre of 10^7^ TCID_50_/ml (**a**), a medium titre of 10^8^ TCID_50_/ml (**b**) and a high titre of 1.4×10^9^ TCID_50_/ml (**c**). The difference between both groups (urban vs. peri-urban) was analysed with the Fisher exact test (ns, *P*>0.05; *, *P*<0.05; **, *P*<0.005). Mosquitoes were harvested at 14 days post-infection for ZIKV.

Urban and peri-urban mosquitoes were infected with low, medium and high titres of a ZIKV strain of the African lineage, isolated in Senegal. ZIKV was able to infect, disseminate and be transmitted by mosquitoes from both urban and peri-urban sites at all titres tested (Table S2). For mosquitoes infected with the medium titre, no significant differences in the infection rate (IR), dissemination rate (DR), transmission rate (TR) and transmission efficiency (TE) were observed between urban and peri-urban mosquitoes (Fig. 4b and Table S2, *P*>0.05). For the low titre blood meal, the DR was significantly different between urban and peri-urban mosquitoes, whereas for the high titre blood meal, a significant difference was found for the IR (Fig. 4a–c and Table S2).

**Fig. 5.**
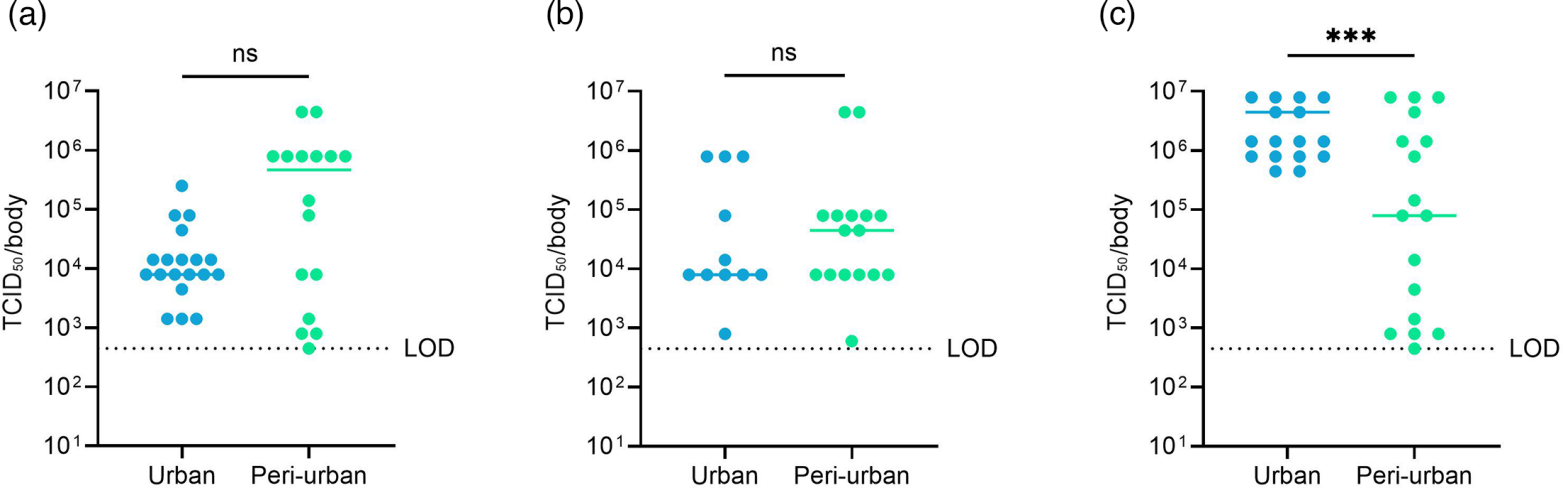
ZIKV (West African strain) infectious titres in the bodies of *Ae. aegypti* infected with a low (**a**), medium (**b**) or high (**c**) virus titre. The viral loads were quantified by endpoint titrations on Vero E6 cells. Each dot on the plot represents an individual mosquito. Mosquito samples were collected at 14 days post-infection. The horizontal lines show the median value. LOD is the limit of detection. Statistical analysis was performed with the Mann–Whitney test; ns, *P*>0.05; ***, *P*<0.001.

For mosquitoes infected with the low virus titre, the median viral loads in the body were 7.9×10^3^ TCID_50_/body (95% CI: 7.9×10^3^–1.4×10^4^) and 4.7×10^5^ TCID_50_/body (95% CI: 1.4×10^3^–7.9×10^5^) for mosquitoes of the urban and peri-urban sites. For the medium inoculum, the median viral loads in the bodies were 7.9×10^3^ TCID_50_/body (95% CI: 7.9×10^3^–7.9×10^5^) and 4.5×10^4^ (95% CI: 7.9×10^3^–7.9×10^4^). For the high titre, the median titre was 4.5×10^6^ TCID_50_/body (95% CI: 7.9×10^5^–4.5×10^7^) and 7.9×10^4^ TCID_50_/body (95% CI: 1.4×10^3^–1.4×10^6^), respectively, for mosquitoes of the urban and peri-urban sites (Fig. 5a–c). For the low and medium titres, no significant difference was found in the viral titres of the mosquito bodies from the urban and peri-urban sites (Fig. 5a–c; *P*>0.05). In contrast, for mosquitoes infected with the high virus titre, the viral loads were significantly different in the bodies of urban and peri-urban mosquitoes (Fig. 5c, *P*<0.001).

**Fig. 6.**
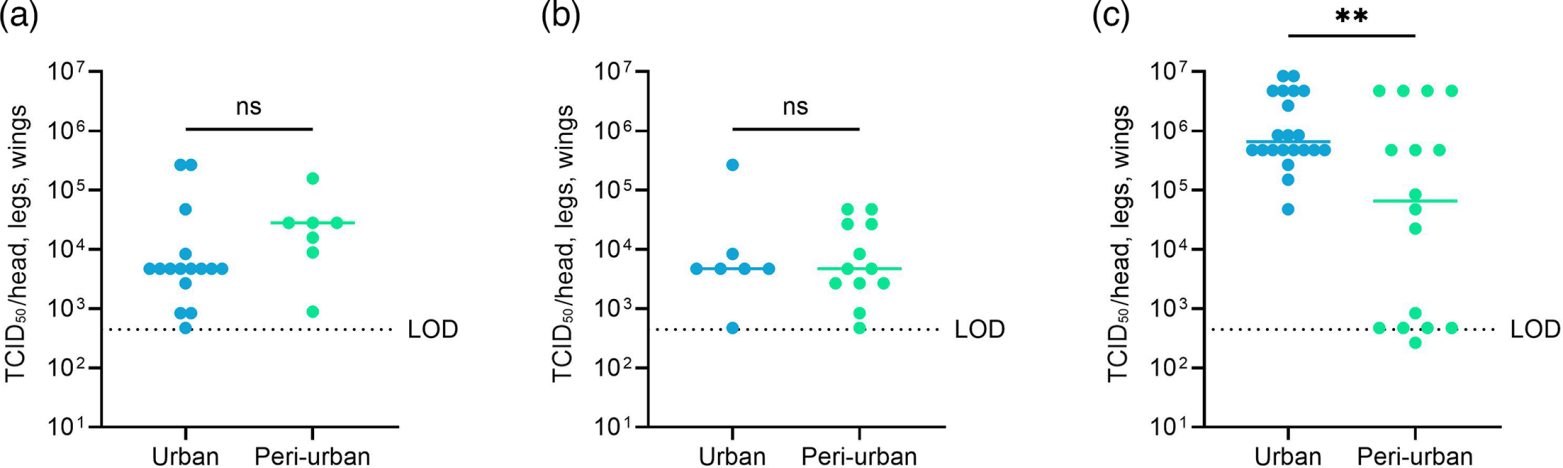
ZIKV (West African strain) titres in heads/legs/wings of *Ae. aegypti* infected with low (**a**), medium (**b**) and high (**c**) virus titres. The viral loads were quantified by endpoint titrations on Vero E6 cells. Each point on the plot represents an individual mosquito. Mosquito samples were collected at 14 days post-infection. The horizontal lines show the median value. LOD is the limit of detection. Statistical analysis was performed with the Mann–Whitney test; ns, *P*>0.05; **, *P*<0.005.

Also in the heads/legs/wings (HLW), a significant difference was observed in the viral loads between urban and peri-urban mosquitoes infected with high virus titre (Fig. 6c), whereas no significant difference was found for the low and medium titres (Fig. 6a, b). For the low titre, a median viral load of 4.7×10^3^ (95% CI: 2.7×10^3^–8.4×10^3^) and 2.8×10^4^ (95% CI: 8.9×10^2^–1.6×10^5^) TCID_50_/HLW was detected in mosquitoes from the urban and peri-urban sites. Mosquitoes infected with the medium titre had median viral loads of 4.7×10^3^ (95% CI: 4.7×10^2^–2.7×10^5^) and 4.7×10^3^ (95% CI: 2.7×10^3^–2.7×10^4^) TCID_50_/HLW (Fig. 6b). In mosquitoes infected with the high virus titre, the median viral loads in HLW were 6.6×10^5^ (95% CI: 4.7×10^5^–4.7×10^6^) and 6.6×10^4^ TCID_50_/HLW (95% CI: 4.7×10^2^–4.7×10^6^) for urban and peri-urban mosquitoes (Fig. 6c).

**Fig. 7.**
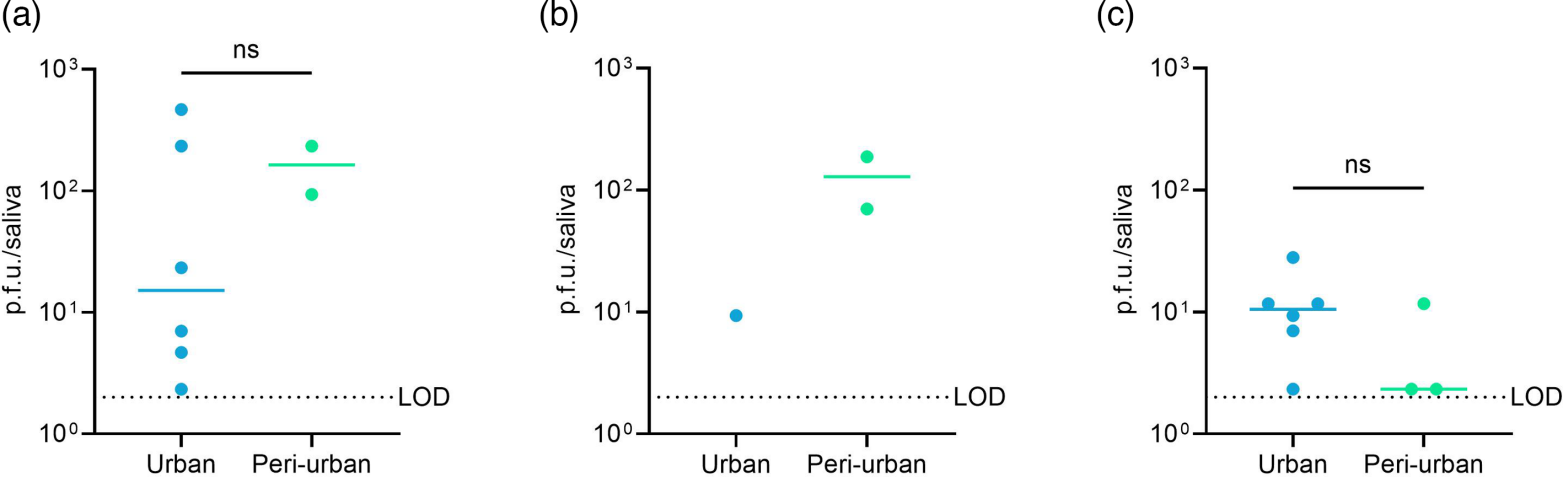
ZIKV (West African strain) titres in the saliva of *Ae. aegypti* infected with low (**a**), medium (**b**) and high (**c**) virus titres. The ZIKV titres were quantified by plaque assay. Each point on the plot represents an individual mosquito. Mosquito samples were collected at 14 days post-infection. The horizontal lines show the median value. LOD is the limit of detection. Statistical analysis was performed with the Mann–Whitney test; ns*, P*>0.05.

Infectious particles were detected in the saliva of mosquitoes infected with the three virus titres. The number of infectious virus particles in the saliva varied from 2.3 to 467 p.f.u./saliva. For the low inoculum, the median viral loads were 15.2 p.f.u./saliva (95% CI: 2.3–467) and 163 p.f.u./saliva (95% CI: 93–233) for urban and peri-urban mosquitoes (Fig. 7a). For the medium inoculum, the viral load was 9.3 p.f.u./saliva for the one urban mosquito that transmitted ZIKV and a median of 129 p.f.u./saliva (95% CI: 70–187) for peri-urban mosquitoes (Fig. 7b). In mosquitoes infected with the high inoculum, the median viral loads were 10.5 (95% CI: 2.3–28) and 2.3 (95% CI: 2.3–12) p.f.u./saliva for urban and peri-urban mosquitoes (Fig. 7c). No significant difference in the saliva titres was found between urban and peri-urban mosquitoes for the low and the high inoculum (Fig. 7a, b).

### *Ae. aegypti* mosquitoes from Burkina Faso showed lower susceptibility to Zika virus from the Asian lineage compared to the African Zika virus strain

**Fig. 8.**
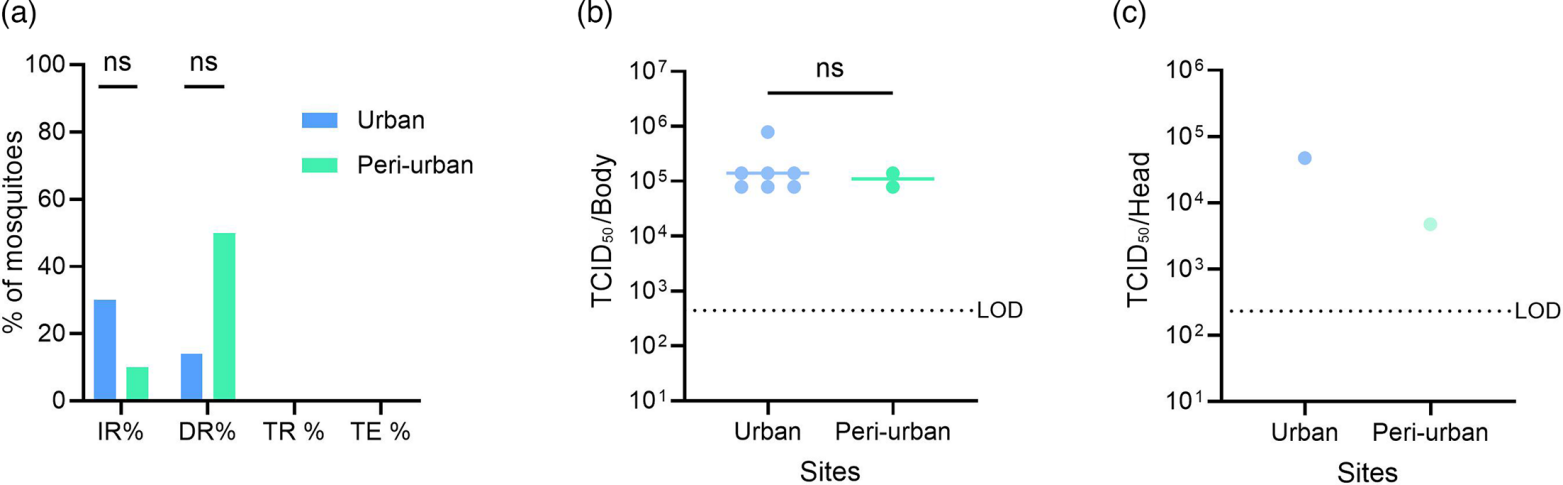
(**a**) Susceptibility of urban (*n*=23) and peri-urban (*n*=20) *Ae. aegypti* for the ZIKV Puerto Rico strain (1×10^8^ TCID_50_/ml). Mosquito samples were collected at 14 days post-infection. The difference between the two groups (urban vs. peri-urban) was analysed with the Fisher exact test (ns, *P*>0.05). (**b–c**) Viral titres in the body (**b**) and in the head/legs/wings of individual mosquitoes (**c**). ZIKV lttres were quantified by endpoint titration on Vero E6 cells. Each point on the plot represents an individual mosquito. The horizontal lines show the median value. LOD is the limit of detection. Statistical analysis was performed with the Mann–Whitney test; ns, *P*>0.05.

Finally, we infected urban and peri-urban mosquitoes with the ZIKV Puerto Rico strain, an epidemic strain from the Asian lineage. *Ae. aegypti* from both sites did not become infected with the low virus titre. In contrast, after feeding on a blood meal with the medium virus titre, mosquitoes became infected and disseminated the virus (Table S3), indicating that the susceptibility to the Asian ZIKV strain was titre-dependent. However, mosquitoes from both sites were not able to transmit ZIKV. No significant differences were found in the infection and dissemination rates between mosquitoes of both sites (Fig. 8a). In comparison to the African ZIKV strain (Fig. 4), lower infection and dissemination rates were observed with ZIKV of the Asian lineage.

Infectious virus was detected in the bodies, and in one heads, wings and legs sample per site, but not in saliva. The median titre in the bodies was 1.4×10^5^ (95% CI: 7.9×10^4^–7.9×10^5^) and 1.1×10^5^ (95% CI: 7.9×10^4^–1.4×10^5^) TCID_50_/body for mosquitoes of the urban and peri-urban sites, respectively (Fig. 8b). In the wings, legs and heads, the virus titre was 4.7×10^4^ TCID_50_/HLW for one urban mosquito and 4.7×10^3^ TCID_50_/HLW for one peri-urban mosquito (Fig. 8C).

## Discussion

Previous research on *Ae. aegypti* mosquitoes from Burkina Faso has primarily focused on their bio-ecology and insecticide resistance [30,33], while the number of studies investigating the vector competence of local mosquito populations has been limited. Although earlier studies have confirmed the presence of the chikungunya virus in *Ae. aegypti* mosquitoes from Burkina Faso [34,35], and serological studies have identified ZIKV antibodies in humans, the intrinsic capability of the local mosquito populations to become infected and transmit chikungunya virus or ZIKV had not been thoroughly examined.

In this study, we report the vector competence of *Ae. aegypti* mosquitoes collected in urban and peri-urban sites in the capital of Burkina Faso for CHIKV and ZIKV. Mosquitoes from Burkina Faso failed to transmit CHIKV when infected with a low virus titre in the blood meal. However, when infected with the medium virus titre, CHIKV was detected in saliva, showing that urban and peri-urban mosquitoes were able to transmit CHIKV. These results are in line with a study from Kenya where CHIKV transmission was shown to be titre-dependent [36]. Another recent study from Senegal reported a similar infection rate for *Ae. aegypti* from Kedougou at 5 days post-infection when infected with a titre of 10^7^ p.f.u. ml^−1^ [37]. However, in contrast to our results, one mosquito was found to disseminate the virus and transmit it. In a study from Central Africa, the reported transmission rates were lower compared to the transmission rates observed in this study [38].

The vector competence results of this study thus align with the recent detection of human CHIKV infections in Burkina Faso [26] and the detection of CHIKV antibodies in human sera [25]. The modelling results reported in the latter study suggested that several unreported epidemics swept through the country over 40 years. The titre dependence of *Ae. aegypti* from Burkina Faso to become infected with CHIKV and the low transmission rates might explain the limited circulation of CHIKV observed in the country in recent years.

Both populations of *Ae. aegypti* from Ouagadougou were able to transmit the Senegal strain of ZIKV under laboratory conditions, suggesting that mosquitoes from urban and peri-urban sites are competent in transmitting this virus. These findings are consistent with previous studies on vector competence, indicating that African *Ae. aegypti* can indeed efficiently transmit ZIKV [20,37, 39, 40]. Interestingly, a differential susceptibility of *Ae. aegypti* mosquitoes to ZIKV strains from the African and Asian lineages has been reported by several studies. For instance, mosquitoes from Colombia demonstrated a higher transmission efficiency for African ZIKV strains compared to Asian strains [41]. Similar results were observed for African *Ae. aegypti* populations, with higher susceptibility to African strains compared to Asian strains [20]. In this study, we observed a similar pattern, with lower infection and dissemination rates for the ZIKV strain of the Asian lineage compared to the African lineage strain in both urban and peri-urban mosquito populations of Ouagadougou.

Interestingly, it has been suggested that for African *Ae. aegypti* populations, the susceptibility to ZIKV is correlated with the level of domestication of the mosquito. The domestic subspecies *Aaa* is characterized by light scaling on the back of the abdomen (first tergite), while the *Aaf* subspecies has dark scaling at this site [42]. The light scaling on the back of the abdomen has recently been shown to be strongly correlated with the preference for human odours [20]. The *Ae. aegypti* population of Ouagadougou was found to be one of the West African populations that did not show a preference for human or animal odours, unlike most African populations that had a clear animal preference. Additionally, the percentage of light scaling in the Ouagadougou population was increased, indicating a certain degree of domestication. We recently confirmed this finding, showing that an *Ae. aegypti* mosquito population from the urban site of Ouagadougou exhibited more light scaling than the mosquito population from the peri-urban site [43].

## Conclusion

This study reports for the first time the vector competence of *Ae. aegypti* mosquito populations from Ouagadougou in Burkina Faso for ZIKV and CHIKV. Both urban and peri-urban populations of *Ae. aegypti* were competent for CHIKV and the African lineage of ZIKV in laboratory settings. The susceptibility of CHIKV transmission was dependent on the virus titre in the blood meal, while this was not observed for the African ZIKV strain. In contrast, transmission of ZIKV from the Asian lineage by *Ae. aegypti* from Ouagadougou was not observed. These findings underscore the susceptibility of *Ae. aegypti* from Ouagadougou to native African strains of CHIKV and ZIKV, emphasizing the significance of arbovirus surveillance to mitigate potential outbreaks in the region.

## Supplementary material

10.1099/jgv.0.002103 Uncited Supplementary Material 1.

## Abbreviations

*Aaa*: *Aedes aegypti aegypti*
*Aaf*: *Aedes aegypti formosus*
CHIKV: chikungunya virus
DENV: dengue virus
DR: dissemination rate
ECSA: East Central South African
HLW: head/legs/wings
IR: infection rate
1200LG: 1200 logements
MEM: minimum essential medium
NEAA: non-essential amino acids
PenStrep: penicillin-–streptomycin
TE: transmission efficiency
TR: transmission rate
ZIKV: Zika virus
