# Supplementary material for: Aedes aegypti mosquitoes from Burkina Faso can transmit the chikungunya virus and Zika virus of the African lineage, but not the Zika virus of the Asian lineage
Source: J Gen Virol. 2025 Jun 19;106(6):002103. doi: 10.1099/jgv.0.002103 (PMC12179391; doi:10.1099/jgv.0.002103)
Supplement: Uncited Supplementary Material 1. [file jgv-106-02103-s001.pdf]

Supplementary Information

Table 1. Infection, dissemination and transmission rates and efficiency for Chikungunya virus in *Ae. aegypti* infected with different virus titers in the blood meal.

Virus titer in blood meal (PFU/mL)	Collection sites	% Infection[1]	% Dissemination[2]	% Transmission[3]	% Transmission efficiency[4]
$1x10^7$	Urban	35 (7/20)	0 (0/20)	-	-
	Peri-urban	0 (0/30)	-	-	-
$2x10^8$	Urban	65 (13/20)^{ns}	84.6 (11/13)*	18.2 (2/11)^{ns}	10 (2/20)^{ns}
	Peri-urban	63 (17/27)^{ns}	41.2 (7/17)*	14.3 (1/7)^{ns}	3.7 (1/27)^{ns}

[1]Percentage mosquitoes containing the virus in their bodies (number of positive bodies/total tested).
[2]Percentage mosquitoes containing the virus in their heads, wings, and legs (number of positive head/leg/wing samples per total tested).
[3]Percentage mosquitoes containing the virus in their saliva (number of positive saliva samples per total tested).
[4]Percentage of mosquitoes containing the virus in their saliva (number of positive saliva samples per total tested)
ns = not significantly different and significantly different: (= p<0.05, ** = p<0.005, *** = p<0.0001; Fisher exact test)*

Table 2. Infection, dissemination and transmission rates and efficiency for Zika virus in *Ae. aegypti* infected with different titers of virus in the blood meal.

Virus titers in blood meal (TCID50/mL)	Collection sites	% Infection[1]	% Dissemination[2]	% Transmission[3]	Transmission efficiency[4]
$1x10^7$	Urban	79.2 (19/24)^{ns}	84.2 (16/19)*	37.5 (6/16)^{ns}	25 (6/24)^{ns}
	Peri-urban	65.2 (15/23)^{ns}	46.7 (7/15)*	28.6 (2/7)^{ns}	8.7 (2/23)^{ns}
$1x10^8$	Urban	64.7 (11/17)^{ns}	63.6 (7/11)^{ns}	9.1 (1/11)^{ns}	5.9 (1/7)^{ns}
	Peri-urban	53.3 (16/30)^{ns}	68.7 (11/16)^{ns}	12.5 (2/16)^{ns}	6.7 (2/30)^{ns}
$1.4x10^9$	Urban	100 (22/22)**	100 (22/22)^{ns}	27.3 (6/22)^{ns}	27.3 (6/22)^{ns}
	Peri-urban	70.8 (17/24)**	94.1 (16/17)^{ns}	18.7 (3/16)^{ns}	12.5 (3/24)^{ns}

[1]Percentage of mosquitoes containing the virus in their bodies (number of positive bodies/total tested).
[2]Percentage mosquitoes containing the virus in their wings and legs (number of positive leg/wing samples per total tested).
[3]Percentage mosquitoes containing the virus in their saliva (number of positive saliva samples per total tested).
[4]Percentage of mosquitoes containing the virus in their saliva (number of positive saliva samples per total tested).
*ns = not significantly different and significantly different: * = p<0.05, ** = p<0.005, *** = p<0.0001 (Fisher exact test)*

Table 3. Infection, dissemination and transmission rates and efficiency for the Zika virus epidemic strain (PRVABC59) in *Ae. aegypti* infected with different titers of virus in the blood meal.

Virus titers in blood meal (TCID50/mL)	Collection sites	% Infection[1]	% Dissemination[2]	% Transmission[3]	% Transmission efficiency[4]
$1x10^7$	Urban	0 (0/20)	-	-	-
	Peri-urban	0 (0/18)	-	-	-
$1x10^8$	Urban	30.4 (7/23)^{ns}	14.28 (1/7) ^{ns}	-	-
	Peri-urban	10 (2/20)^{ns}	50 (1/2) ^{ns}	-	-

[1]Percentage mosquitoes containing the virus in their bodies (number of positive bodies/total tested).
[2]Percentage mosquitoes containing the virus in their wings and legs (number of positive leg/wing samples per total tested).
[3]Percentage mosquitoes containing the virus in their saliva (number of positive saliva samples per total tested).
[4]Percentage of mosquitoes containing the virus in their saliva among the total tested mosquitoes)
*ns = not significantly different and significantly different: * = p<0.05, ** = p<0.005, *** = p<0.0001 (Fisher exact test)*
